# Investigation of Coatings, Corrosion and Wear Characteristics of Machined Biomaterials through Hydroxyapatite Mixed-EDM Process: A Review

**DOI:** 10.3390/ma14133597

**Published:** 2021-06-28

**Authors:** Md Al-Amin, Ahmad Majdi Abdul-Rani, Mohd Danish, Saeed Rubaiee, Abdullah bin Mahfouz, Harvey M. Thompson, Sadaqat Ali, Deepak Rajendra Unune, Mohd Hafis Sulaiman

**Affiliations:** 1 Department of Mechanical Engineering, Universiti Teknologi Petronas, Seri Iskandar 32610, Malaysia; 2 Department of Mechanical and Materials Engineering, University of Jeddah, Jeddah 21589, Saudi Arabia; mdanish@uj.edu.sa (M.D.); salrubaiee@uj.edu.sa (S.R.); 3 Department of Industrial and Systems Engineering, University of Jeddah, Jeddah 21589, Saudi Arabia; 4 Chemical Engineering Department, University of Jeddah, Jeddah 21589, Saudi Arabia; asbinmahfouz@uj.edu.sa; 5 School of Mechanical Engineering, University of Leeds, Leeds LS2 9JT, UK; h.m.thompson@leeds.ac.uk; 6 School of Mechanical & Manufacturing Engineering, National University of Sciences and Technology (NUST), H-12, Islamabad 44000, Pakistan; sadaqat.ali@smme.nust.edu.pk; 7 Department of Materials Science and Engineering, INSIGNEO Institute of in Silico Medicine, University of Sheffield, Sheffield S1 3BJ, UK; d.unune@sheffield.ac.uk; 8 Department of Materials Science and Engineering, Kulliyah of Engineering, International Islamic University Malaysia, Kuala Lumpur 50728, Malaysia; hafisulaiman@iium.edu.my

**Keywords:** hydroxyapatite, electro-discharge, biomaterials, coatings, corrosion, wear

## Abstract

Together, 316L steel, magnesium-alloy, Ni-Ti, titanium-alloy, and cobalt-alloy are commonly employed biomaterials for biomedical applications due to their excellent mechanical characteristics and resistance to corrosion, even though at times they can be incompatible with the body. This is attributed to their poor biofunction, whereby they tend to release contaminants from their attenuated surfaces. Coating of the surface is therefore required to mitigate the release of contaminants. The coating of biomaterials can be achieved through either physical or chemical deposition techniques. However, a newly developed manufacturing process, known as powder mixed-electro discharge machining (PM-EDM), is enabling these biomaterials to be concurrently machined and coated. Thermoelectrical processes allow the migration and removal of the materials from the machined surface caused by melting and chemical reactions during the machining. Hydroxyapatite powder (HAp), yielding Ca, P, and O, is widely used to form biocompatible coatings. The HAp added-EDM process has been reported to significantly improve the coating properties, corrosion, and wear resistance, and biofunctions of biomaterials. This article extensively explores the current development of bio-coatings and the wear and corrosion characteristics of biomaterials through the HAp mixed-EDM process, including the importance of these for biomaterial performance. This review presents a comparative analysis of machined surface properties using the existing deposition methods and the EDM technique employing HAp. The dominance of the process factors over the performance is discussed thoroughly. This study also discusses challenges and areas for future research.

## 1. Introduction

In material science, a biomaterial is characterized as a matter designed to take a shape that is used, alone or as a part of complex method, to guide the direction of any diagnostic or therapeutic technique by regulating interactions with components of living systems [1]. Generally, biomaterials are different from ordinary materials as regards their applications. Biomaterials constructed from the metallic materials were first introduced in 1969 at Clemson University, Clemson, SC, USA. These materials are commonly applied for manufacturing bio-implants such as dental, orthopedic, heart-valves, artificial-hearts, and vascular-grafts that are routinely used to restore damaged tissues and living organs in the body. Due to special applications of biomaterials, these materials must have excellent biocompatibility, biofunctions, high mechanical strength, and superior resistance to wear and corrosion [2,3,4,5]. Although there are three kinds of biomaterials, including metallic, natural and synthetic polymers, and ceramics, available for biomedical applications, approximately 70–80% of bio-implants are made from metallic biomaterials, owing to their high mechanical strength, stiffness, and long durability; as reported in the literature [4,5,6,7]. Table 1 shows the mechanical properties, biocompatibility, and corrosion resistance of commonly employed biomaterials in biomedical applications. Steel-alloy, titanium and its alloy, magnesium and its alloy, cobalt-based alloy, titanium-zirconium based alloy, zirconium and molybdenum-based alloy, and noble metallic alloys are known as metallic biomaterials. An upward trend of metallic biomaterial utilization has been observed due to dramatic increases in the aging population, bone diseases, and accidents [8,9,10].

Although the metallic biomaterials have higher mechanical strength compared to other biomaterials, these materials are inappropriate to apply directly in the living body due to their releasing toxic particles (Ni^2+^, V^3+^, Cr^3+^, Mo^2+^, and so on) and exhibiting poor bioactivity [12,13,14,15]. Failure of 316L steel-based implants has been reported to occur because of corrosion (41%), fatigue (25%), impurities (17%), wear (7%), and bacterial infection (10%), which are surface dependent issues [16]. Surface modification is therefore proposed as a key solution to resolve these limitations. The machined surfaces prepared by the conventional machining processes such CNC milling, CNC lathe, turning, shaping, boring, and so on do not ensure the biocompatibility and the sustainability of biomaterials [17]. The existing surface coating technologies, such as chemical-vapor-deposition (CVD), physical-vapor-deposition (PVD), sol-gel, plasma spraying, laser-surface-melting, electrochemical-deposition, spray-pyrolysis, electrophoretic-deposition, dip-coating, and hybrid form of sol-gel and dip coating, are capable of forming a biocompatible coating on the biomaterials [18]; whereas these techniques show some limitations, as they are incapable of forming a surface without micro-cracks and thermal stresses, unable to form a nano-porous surface, and unable to shape and form the coating simultaneously, as well as require high processing cost [19].

Powder mixed-electro discharge machining (PM-EDM) is a newly developed and innovative manufacturing process which was first implemented in 1980. Researchers have recently focused on enhancing the machining efficiency and the characteristics of modified biomaterial surface through a novel process called PM-EDM, for commercial implementation [20,21,22,23]. During this process, suitable electrically conductive or moderate-conductive particles are suspended in a dielectric liquid to improve the machining performance and enhance the machined surface characteristics, which makes this process different from the conventional EDM process [22,24,25,26,27]. Since the PM-EDM technique follows thermo-electrical process like EDM, a huge temperature range, from 8000 °C to 12,000 °C, is produced due to creating successive electrical sparks during the machining. Obviously, the temperature generated during the operation is much higher than the fusing point of the employed specimen, electrode, dielectric liquid, and additive powders, resulting concurrent removal and deposition of the fused materials on the machined surface [28,29,30]. Hence, the outstanding capabilities of the PM-EDM technique include simultaneous machining capability and modification of the machined surface. This technique shows several advantages over the conventional manufacturing process, including fabrication of complex parts and machining of both the hard and brittle materials regardless of their thickness. The machining performance relies on the corresponding parameters of the PM-EDM process, including the current (A), pulse duration (µs), gap voltage (V), polarity, and added powder concentration (g/L) [8,31,32,33,34,35,36,37]. Demand for the EDM technique has been created in industries due to an increase in dependence on using metallic biomaterials such as steel alloy, titanium alloy, cobalt alloy, Zr-based alloy, and magnesium alloy with high mechanical properties [8,9].

The modified surface responses, such as the coating properties, microhardness, and resistance to wear and corrosion, significantly influence the biocompatibility, biofunctions, and durability of the biomaterials [38,39,40]. Hydroxyapatite (HA) is considered a bio-ceramic powder that is used to form a biocompatible coating on the machined biomaterial surface to enhance the biological response because it serves Ca, P, and O [9,11]. Studies reported a less than 2% bio-implant failure after following up for several years, assuring the clinical success of the HA powder (HAp) in biomedical applications [41,42,43,44,45,46,47]. However, sudden failure of HAp-based bio-implants was reported recently, due to deterioration resulting from faster dissolution of the HAp under body fluid conditions as HAp is brittle in nature and has poor crystallinity [47,48,49,50,51,52]. Researchers have been investigating the effects of a HAp mixed-EDM process on modified surface characteristics over the last decade. Outstanding progress in coating thickness, microhardness, biocompatibility, and corrosion resistance of the treated metallic biomaterials has been claimed in recent studies applying HAp in the EDM process [11,53,54,55,56].

Today, few research studies are available regarding the treated surface characteristics, such as the microhardness, coating features, and wear and corrosion resistance of the metallic biomaterials using the HAp added-EDM process, and the influence of the PM-EDM process factors on the surface characteristics. In addition to this, there is a lack of literature which illustrates in detail the importance of the modified surface response for the biomaterials’ performance. Therefore, this review provides in-depth information for the fundamental sciences regarding the issues to the researchers to use in further studies. In this review article, a comprehensive analysis of the status of the coating features, microhardness, and corrosion and wear behavior of the biomaterials using HAp added-EDM is presented. Furthermore, the importance of the coating, wear, and corrosion properties on the biomaterials’ performance are discussed thoroughly. In this study, a comparative study on the treated biomaterials surface characteristics utilizing both the HAp added-EDM and the existing coating techniques employing HAp is critically discussed. The effects of the associated process parameters on the modified surface properties have been reported thoroughly. The surface topography and morphology of the machined surface have been demonstrated to evaluate the analysis. In this article, the challenges for the PM-EDM process that may lead to future research are also summarized.

## 2. Fundamentals of Surface Modification through the PM-EDM Method

**Figure 1:**
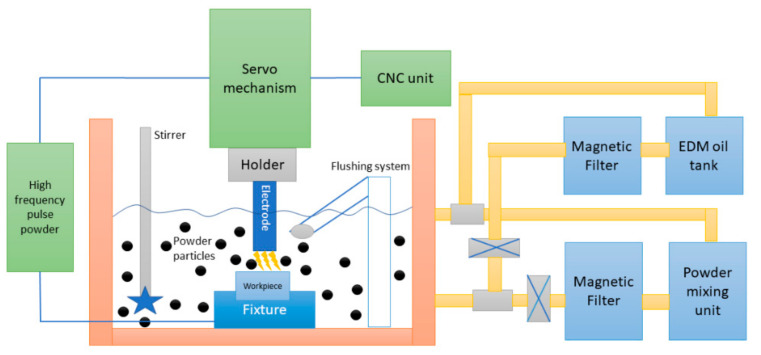
Schematic diagram of the PM-EDM technique reproduced with permission from ref. [57].

The fundamental science of PM-EDM method is still in the research phase, owing to the lack of in-depth clarification and the intricate nature of the associated variables. The selected electrically conductive or moderately conductive powders are amalgamated in the working liquid of the PM-EDM process. Figure 1 shows a model of the PM-EDM technique.

The schematic diagram describes the components and full set up of the PM-EDM technique. When electrical potential ranging from 75 V to 350 V is applied, an electrical magnetic field varying from 10^5^ V/m to 10^7^ V/m is created, retaining a tiny gap between the specimen and the electrode. A plasma path resulting from ionization of the dielectric is created. The plasma channel, electrically conductive in behavior, causes the ions to flow. Due to the collisions of ions in the plasma channel, discrete electrical sparks occur in the discharge channel, resulting in generation of high temperatures, ranging from 8000 °C to 12,000 °C. Because of the elevated temperature, the surrounding particles from the employed specimen, electrode, insulator liquid, and additive powders are fused and eroded. The phase transformation of the added HAp particles with increasing temperature produced during the EDM process are shown in Table 2.

**Figure 2:**
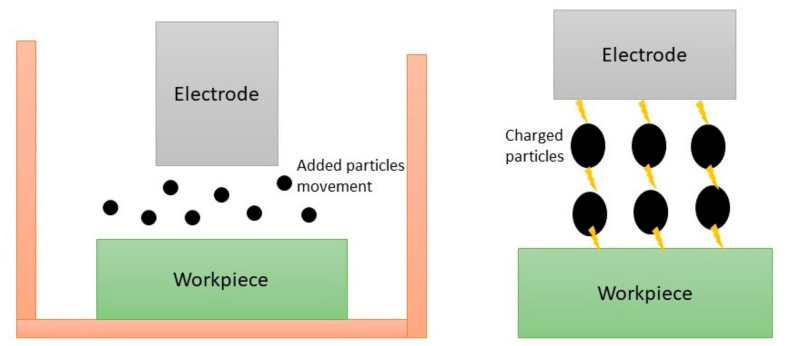
Mechanism of the added particle movement and chain formation reproduced with permission from ref. [57].

Since a very high temperature is generated during the PM-EDM process, the suspended additives such as HAp, CNT, SiC and so on turn into different phases. Gaseous bubbles are produced by the decomposed dielectric liquid, which take the eroded debris away from the machining zone. Owing to the decrease in the compressive stresses on the bubbles, they collapse. The mechanisms of the added particle movement and chain-like formation during the machining are depicted in Figure 2.

**Figure 3:**
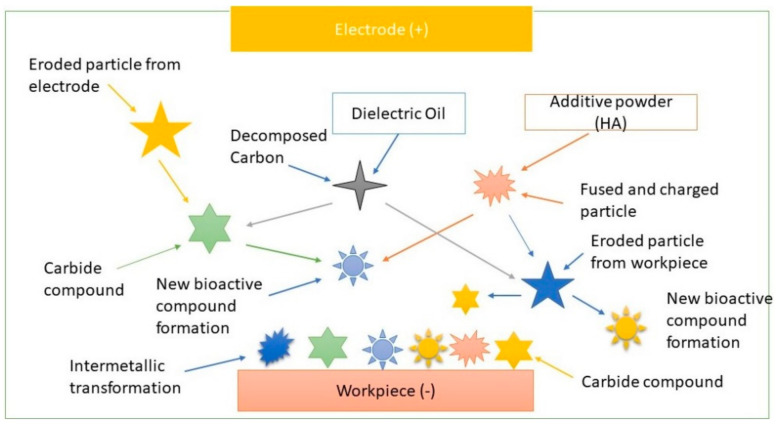
Mechanisms of alloy formation and debris deposition during the powder mixed-EDM (PM-EDM) process reproduced with permission from ref. [57].

The added particles are energized by the attached ions on their surfaces during the discharge condition and pursue a zigzag movement due to the counter attractions of the opposite charges. Owing to the generated magnetic effect and the charged particles in the machining area, a capacitive effect is created, resulting in a chain-like connection among the charged particles. As a result, the insulating strength of the dielectric liquid is decreased. Furthermore, faster and uniform electric sparks are generated in the machining area resulting in an enhanced machining efficiency, such as the material erosion rate [10,19,21,28,37,57,59,60]. The mechanisms of alloy formation and debris deposition during the PM-EDM cycle are demonstrated in Figure 3.

The fundamental mechanisms behind the migration and deposition of the fused materials that are assumed to occur during the PM-EDM technique by melting, chemical reactions, and solidification processes are stochastic in behavior, since both the migration and the deposition of the eroded materials depend on the related parameters and physical characteristics of the added powders, specimen, and electrode. During the machining, the produced thermal energy is high enough to fuse both the electrode’s and the specimen’s surface materials, the suspended powders, and the dielectric. Due to the retainment of a tiny machining void and generating a very high temperature, more materials from both the working-part and the electrode are melted and eroded rather than flushing them. Consequently, some of the eroded debris are accumulated on the machined part. At the same time, due to their going through the narrow discharge gap, the suspended particles in the working liquid that serve as a coolant are partially melted and charged. The formed chain like connections between the charged particles facilitate their rapid deposition on the modified part due to electromagnetic forces, electrophoresis negative pressure, and electrostatic forces. Moreover, the melted and charged particles are attracted by both the ionized debris eroded from the electrodes and the tool surfaces, with the opposite tool polarity leading to oxide formation due to chemical reactions among them. The formed oxide alloys are deposited and solidified on the machined surface. The decomposed working liquid serves C and OH, which creates carbides and oxide alloys. Some of the chemical reactions that may occur during the carbide and oxide formation are mentioned as follows.

Decomposition of HAp (above 15,550 °C):

$$Ca_{10}{\left(PO_{4}\right)}_{6}{\left(OH\right)}_{2}\to \alpha -Ca_{3}{\left(PO_{4}\right)}_{2}+Ca_{4}{\left(PO_{4}\right)}_{2}O+H_{2}+\frac{1}{2}O_{2}$$

Chemical reactions during the oxides, carbides, and intermetallic alloy formation:

Ti+C→TiC

$$Ti+2H_{2}O\;\left(gas\right)\to TiO_{2}+2H_{2}$$

$$TiO_{2}+3C\to TiC+2CO$$

$$Ni^{2+}+Ti^{2+}\to NiTi$$

$$3Ti+\frac{1}{2}O_{2}+Ca_{3}{\left(PO_{4}\right)}_{2}\to 3CaTiO_{3}+2P$$

$$3Cr^{3+}+2C\to Cr_{3}C_{2}$$

Moreover, intermetallic compounds are produced on the machined substrate due to agglomeration of the molten and the charged materials. The cooling process solidifies the molten materials and reconstructs the metallic compounds during the duration of the discharge breakup, which proceeds from the start of the recast layer or coating formation process. However, a rapid cooling process results uneven shrinkage of the formed metal alloys, causing residual stress and micro-crack formation. The gases, such as hydrogen, nitrogen, oxygen, and so on, that are produced during the solidification process are released from the machined surface, resulting nanopores and foamy shaped surface [11,34,48,57,60,61,62].

## 3. Importance of Hydroxyapatite-Based Coatings’ Characteristics on the Biomaterials’ Performance

### 3.1. HAp and Its Influence on the Response of Machined Biomaterials

Biocompatible coating is a crucial prerequisite for biomedical applications, as it provides a stable condition for recovery of the injured tissues by interacting with different naturally formed bioactive materials. The HA formulated by Ca_10_(OH)_2_(PO_4_)_6_ is a source of Ca, P, H, and O, which are the inorganic elements resembling bone. Since HAp has excellent adaptation to in vivo and in vitro aspects, it possesses superior biocompatibility and osseointegration, which lead to regeneration of the hard tissues. Compared to other bio-ceramics, the abatement potentiality of both the flammable impacts and the adverse chemical reactions in the body by utilizing the HAp-based coating is higher [9,48,62,63,64,65]. Consequently, a HAp-based coating is proposed as a perfect candidate for enhancing the biocompatibility, mechanical characteristics, and corrosion resistance of biomaterials. Although HA-based ceramic exhibits a lower Young’s modulus, similar to bone, compared to metallic biomaterials, bio-implants made from HA-based ceramics have proven to be inappropriate for load-bearing conditions because of their poor crystallinity and brittleness [11,40,48,66,67,68,69]. Therefore, HAp is utilized as additive powders for the purpose of coating formation, and which promotes both the mechanical and the biological properties of the biomaterials. The HAp-based coating requirements approved by the FDA, USA are summarized in Table 3. The addition of HAp in the EDM process has been reported to improve the coating properties, hardness, and wear and corrosion resistance of the modified biomaterial [9,56,70,71,72].

For instance, Chander et al. [70] confirmed the formation of oxide and carbide alloys on the modified Ti-based alloy through the HAp mixed-EDM method, which enhanced the coating thickness, microhardness, and corrosion behavior of the treated biomaterial. In the research work by Gurpreet et al. [73], the amalgamation of HAp in the working fluid of the EDM method increased the microhardness of the altered 316 L surface by 160%. In the study by Chander et al. [56], the corrosion resistance and microhardness of the machined Mg-alloy were increased by 90.85% and 1.5 times, respectively, while machining using an HAp mixed-EDM process. Al-Amin et al. [74] ensured a uniform coating on a machined 316L steel surface having a thickness of 15.295 µm through the HAp added-EDM process, which gave excellent biocompatibility and corrosion resistance.

### 3.2. Influence of Coating Phase and Thickness on Biomaterial Performance

Due to insufficient biological responses and the release of toxic elements (Cr, Co, Ti, V, Al, Mo, Fe, Ni, and so on) from attenuated metallic biomaterial surfaces, caused by wear and corrosion actions, these biomaterials are inappropriate for being inserted in the living body directly [19,75,76]. As a consequence, coating the surface with bio-ceramic elements is proposed as an effective method to ensure low degradation of the metallic biomaterials by protecting them from wear and corrosion propagation [77]. An effective hydroxyapatite-based coating can improve bone formability, host responses, microhardness, and resistance to wear and corrosion [78,79,80,81,82,83,84,85,86]. Both the crystalline and the amorphous phase-based coatings of biomaterials influence the biocompatibility, mechanical properties, and corrosion behavior, which were discussed thoroughly in the previous literature reports. Coatings with a high amorphous phase ensure an improvement in the mechanical characteristics, including the microhardness and fatigue strength, whereas layers with high crystalline phase possess a robust coating adhesion strength, which improves the durability of the biomaterials. Moreover, because of amorphous phase in the machined coating, the hardness and the coefficient of friction are observed to improve, causing an improvement in wear resistance property of the biomaterials [87]. The amorphous nano-crystalline phase formation in the coating exhibits poor corrosion potential because of the nano-crystalline phase, which leads towards corrosion propagation, whereas partially crystalline-based layers have been reported to have a higher corrosion resistance compared to fully amorphous coatings [87,88,89]. In crystalline-based coatings, the chemical inhomogeneity of the alloys with galvanic coupling actions is observed, causing a low corrosion resistance. On the other hand, an amorphous coating ensures chemical homogeneity of the alloys, which attenuates the galvanic action, resulting in high corrosion resistance. In addition to this, a reduction of the solvent concentration of the corrosion-resistant particles is observed in crystalline-based coatings, resulting in lower corrosion resistance, which is increased when synthesizing the amorphous alloys [87]. Hence, the coatings with a crystalline phase embedded in the amorphous phase have been proposed to enhance the resistance to corrosion in different applications [87,89]. Numerous approaches, such as HVOF, HVAF, plasma spraying, and laser processing, are utilized to ensure the amorphous phase formation in the coating, and employing different additives, including Zr, Fe, Cr, Mo, Al, Ni, and Cu, which are recognized as the amorphous alloys [88,90,91,92,93,94,95,96,97]. Coating thickness is regarded as a critical factor influencing the biomaterial performance. Coating adhesion strength is considered another critical factor that is increased with decreasing the coating thickness, because of storing few residual stresses and increasing the compressive stress in the formed coating. The residuals stresses are produced in the coating due to rapid quenching and impinging of the melted materials during the EDM process, and are decreased with a declining layer thickness [87,98]. Degradation of the fatigue stiffness of the biomaterial is also observed due to increasing the recast layer thickness [99], whereas a high fatigue strength with excellent biocompatibility of the biomaterials was reported to be attained with the formation of a thin recast layer and stable oxide alloys in the coating [100].

### 3.3. Influence of HAp-Based Coatings on the Modified Biomaterial Response

Optimization of the oxide-based coating thickness is proposed to be effective for controlling the corrosion rate of Mg alloys [101]. The mechanical characteristics, including hardness and strength of the cold sprayed Ti-6Al-4V layer are improved with increasing the thickness of a Ti-6Al-4V-based coating. Moreover, the flexural stresses of the coatings decrease with increasing coating thickness because of interparticle failure, unable to conform with large stress concentrations [102]. A thin film, consisting of hard nitride prepared by the reactive magnetron sputtering method, facilitated the enhancement of the resistance to wear of modified metallic biomaterials [98,103]. It was reported that the corrosion resistance of the coated stainless steel through the hydrothermal deposition technique improved with increasing the ZrO_2_-based coating thickness [104]. Furthermore, another study showed that a polyester based-coating prepared by the dip coating technique improved the resistance to corrosion of Mg alloy when increasing the coating thickness [105]. Lynn and Duquesnay [106] studied the impact of HA-based layer thickness on the fatigue strength of a Ti-6Al-4V alloy deposited by the plasma spray process. In this study, a HA-based coating thickness ranging from 0 to 100 µm did not exhibit an impact on the fatigue strength, whereas an increment of the coating thickness to 150 µm decreased the performance. Aksakal et al. [107] investigated the HAp-based substrate thickness effect on the corrosion behavior of both coated 316L steel and Ti6Al4V by the sol-gel technique. In this study, a coating thickness of 72 µm for the coated 316L steel showed the minimum corrosion resistance, while a HAp-based layer thickness of 40 µm for the coated Ti alloy revealed the maximum corrosion resistance. It was also noted that both the adhesion strength and resistance to corrosion of the treated surfaces reduced with augmenting the coating thickness [106]. In the work by Naofumi et al. [108], the optimal layer thickness of CaTiO3 was determined to investigate the bone tissue response. The CaTiO_3_-based coating was prepared utilizing the magnetron sputtering technique. A CaTiO_3_-based coating thickness of 50 nm showed high biocompatibility and bone regeneration, while a CaTiO_3_-based coating thickness of below 50 nm was inferior regarding the tissue responses. Furthermore, a CaTiO_3_-based coating thickness of 50 nm had a crystalline phase, which would be required to enhance the Ca-P formation on the titanium substrate, because of showing a low dissolution of CaTiO_3_ film, but a coating thickness of below 50 nm was not crystallized with perovskite-type CaTiO_3_ [107,108]. Moreover, degradation of the coatings showing low crystallinity was reported to occur rapidly in the living body, which led to use of a thinner film to resolve these problems [109,110]. The recommended commercial HAp-based coating thickness prepared by the plasma spray technique is around 50 µm, even though the coating is assumed to deteriorate rapidly due to the dissolution process [107,111].

**Figure 4:**
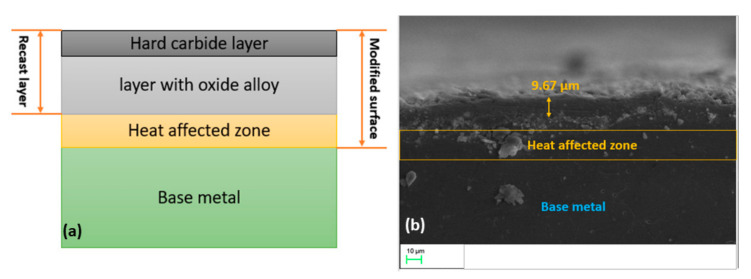
Structure of the modified surface layer: (**a**) illustration of layer, (**b**) obtained layer using the PM-EDM technique (reproduced with permission from ref. [74]).

The recast layer on the treated biomaterials generated by the PM-EDM process comprises three substrates, including a topmost, middle, and base layer. The uppermost portion is typically a very stiff layer, owing to the presence of carbide and intermetallic alloys that are known as a carbide-based substrate. The middle substrate consists of the intermetallic compounds and different types of oxide alloys, which are produced during the machining causing a nanoporous, nanostructured, highly resistant to corrosion, and biocompatible coating formation. The last section is considered a heat-affected region, which is generated during the operation because of the heating and cooling process. The heat affected zone (HAZ) is known to be the non-melted region of the substrate which, as a result of being exposed to high temperatures, has alterations of the material properties [112]. Figure 4 shows the formed recast layer and practically obtained layer during the PM-EDM process. Furthermore, thermal stress is induced in the heat-affected zone due to the thermal mismatch, resulting in mechanical failure [102]. Bui et al. [113] synthesized an antibacterial layer on the treated titanium alloy with the application of nano-Ag powders using the EDM technique. The formed Ag-based coating with a thickness of 2.49 µm improved the microhardness of the coated surface to 528.39 HV. Chander et al. [114] explored the enhancement of modified Ti-35Nb-7Ta-5Zr β-Ti surface characteristics, which was machined through the nano-silicon particles mixed-EDM method. Due to the oxides such as TiO_2_, TiC, Nb_2_O_5_, SiO_2_, SiC, and ZrO_2_ formed in the produced recast layer having a thickness of 15–20 µm, an improvement in biocompatibility, microhardness, corrosion and wear resistance of the modified surface was noticed. In the research conducted by Chander et al. [79], owing to a 15-µm thick recast layer comprised of carbide and oxide, a stiff layer of 1080 HV and high biocompatibility was reported. Aliyu et al. [54] confirmed an improvement in coating adhesion strength and biocompatibility of treated Zr-based bulk-metallic-glass (BMG) due to formation of Ca, P, O, ZrO, and CaZrO_3_ in the coating with a thickness of 23 µm, when HAp was suspended in EDM-oil. However, a thick recast layer was reported to increase the thermal and residual stresses, which may reduce the coating adhesion and cause mechanical malfunctions [87,98,102].

## 4. Influence of Wear Behavior and Microhardness on Biomaterial Performance

**Figure 5:**
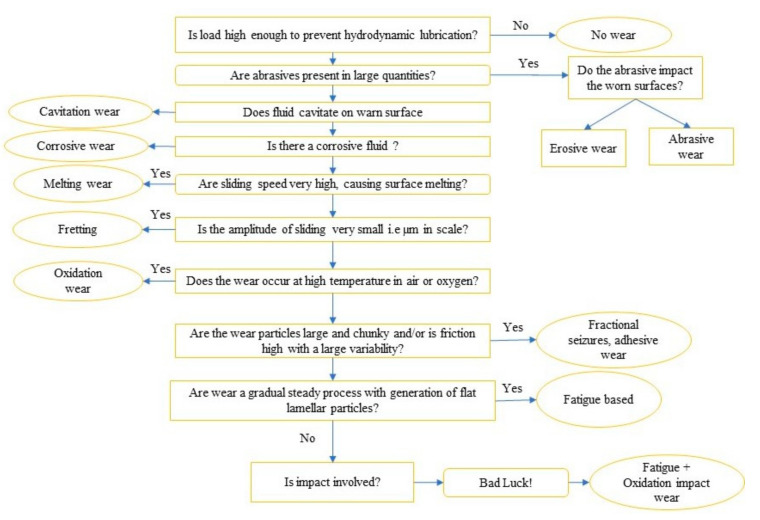
Mechanisms of biomaterial wear behaviour in different conditions (reproduced with permission from ref. [120].

Wear is an important surface property which assists in determining the proper biomaterial selection and bio-implant design. To control and ensure long-term sustainability of the metallic biomaterials, the wear property is considered a major issue that causes materials’ removal from the eroded surfaces while undergoing relative sliding between two or more acting surfaces [79,115,116]. For the synovial joints including entire hip joints, knee, shoulder, and orthopedic implants, wear is considered as a critical issue, because of undergoing critical loads during regular movements, which depend on several parameters, such as material selection; coefficient of friction; contact stress; and surface characteristics, including roughness, hardness, and wettability (lubrication). Localized nano-regions of strong plastic deformation, nano-spall, and the attached spherical elements define the fretting wear that creates tiny and deep cracks on the acting surface, resulting in fatal metallic biomaterial failure [117,118,119]. The mechanisms of biomaterial wear propagation for different conditions are depicted in Figure 5.

This figure provides information regarding the wear propagation when the implanted biomaterial is introduced to a high load, abrasive and corrosive fluids, high sliding speed, a high temperature in the presence of air, and large size debris. Microhardness is another surface property influencing the wear resistance of the biomaterials, but few previous studies have reported an increase in wear resistance due to improving the surface wettability (lubrication) rather than the hardness [121,122,123,124]. The coefficient of friction of a surface, which is defined as a function of the ratio of the friction forces and the normal loads, depends on not only these factors but also on the material characteristics and surface roughness. An improvement in the wettability of the biomaterial surface enhances the lubrication action, decreasing the friction coefficient and increasing the wear resistance [125,126], whereas a rough surface results in an inferior wear resistance, along with a large coefficient of friction [127]. A decline in wear rate is found with a high microhardness and low friction coefficient due to offering a high normal load [123,125]. A high resistance to wear and low friction coefficient are therefore preferable for implants. A greater strain hardening was also proposed to have an impact on improving wear resistance behavior [128,129]. The biomaterial surface therefore must be hard enough to resist the occlusion forces. A modified surface having a hardness of less than 125 kgf/mm^2^ (HV) was reported to have a high tendency to wear the teeth, while a surface having a hardness greater than 340 kgf/mm^2^ (HV) was reported to wear the opposing surfaces [130]. Although the mechanism of wear propagation is complicated, the knowledge of modified surface microstructure was proposed as an essential aspect for predicting the mechanisms of wear propagation. The tribological behavior of a metallic alloy is believed to be governed by the properties of the counteracting surfaces. Moreover, the environment, such as the wetness and dryness, in which two surfaces interact by sliding governs the tribological performance. The operating environment fundamentally determines the implant wear generation, such as the acting loads and characteristics such as one-way slipping, reciprocating, spinning, impact-loads, momentum, and temperature. In recent studies, the presence of a large amount of hard carbides in the formed coating on a Cobalt-based alloy was reported as a source of wear propagation [86,122,131,132,133].

Mechanical wear is proposed to be the primary cause of degradation of biomaterials during the wear process. The fundamental wear mechanisms of implant degradation are identified as abrasion, adhesion, fatigue, and corrosion. In addition, a third body wear is caused by the hard-eroded debris resulting from the reduction of wear resistance [79,122,134,135]. As implants can protect against wear damage in dry environmental conditions, the tribological performance of biomaterials are therefore measured in a simulated body fluid condition. After the implantation of implants in the body, they have to tolerate various harsh environments, such as high salinity, organic elements, acids, and fluorides, depending on the application. Consequently, an oxide-based coating deteriorates in these conditions, and the implants experience extreme breakdown with escaping metal ions, due to an inability for re-passivation. During wear propagation, the amount of overall mass loss is calculated by the sum of elemental loss occurring from both the mechanical wear and the wear accelerated corrosion; however, the wear accelerated corrosion forms a small percentage of the total amount [117,122,136,137]. Wear which is the primary cause of the implant failure has a great influence on the biomaterial performance when these are inserted into the human body [130]. Due to the wear process, the metallic debris having a size of less than 0.05 µm are ejected from the articulating surfaces of the inserted metallic biomaterials, which can vary based on the applied materials [86,138,139]. The eroded debris act as foreign particles, which can be dissolved in the body, resulting in inflammatory responses with the formation of pseudo-tumors, implant loosening, osteolysis, periprosthetic bone destruction, hypersensitivity (metal allergy), and carcinogenicity. The wear behavior therefore reduces the biological response and life period of bio-implants, which may result in revision surgery. Most importantly, around 4–5% of bio-implant failure, inserted within 6–7 years, has been reported to be due to wear debris generation [138,140,141,142,143,144,145].

## 5. Importance of HAp-Based Coatings for the Microhardness and Wear Behavior of Biomaterials

To resolve these problems, researchers are concentrating on alteration of the biomaterial surface to obtain a surface with the attributes of high biocompatibility, corrosion and wear resistance, and good mechanical characteristics. An appropriate coating formation not only enhances the wear resistance but also increases the soundness of the joints implanted. Furthermore, the wear and coefficient of friction decrease when applying an appropriate coating method, and the surface hardness and lubrication are improved as well [86,125]. Yuichi et al. [146] explored the influence of delamination of an HAp-based coating prepared using the plasma spray method on the fretting wear. An improvement in the wear behavior of the HAp-based coating was recorded due to delamination, which increased the relative slip amplitudes. Melanie et al. [147] investigated the impact of an HAp-based coating on the resistance of wear element migration caused by releasing the wear particles in the body and osteolysis. A significant improvement in the prevention of migration of the interfacial wear particles was reported, which resulted in a reduction in the osteolysis effects. In the research work of Reza et al. [148], the friction coefficient of a HAp-based coated Ti-6Al-4V surface using the thermal plasma spray technique showed a downward trend, which caused a high wear resistance. Furthermore, the fatigue behavior of the coated surface was improved. However, the application of HAp-based coatings was recently claimed to be limited, as it contributes to poor mechanical characteristics such as wear resistance, fatigue, microhardness, and rapid dissolution behavior, which led to the addition of reinforcement additives such as Sr, CNT, TiO_2_, Al_2_O_3_, and so on to HAp [148,149,150,151].

Gurpreet et al. [152] explored the microhardness and wear behavior of coated 316L steel prepared through the TiO_2_ mixed-EDM process. The results showed an increase in microhardness by 233% and a superior wear resistance that was increased by 80%. The formation of titanium perovskite on a modified Ti-6Al-4V surface prepared using the calcium chloride mixed-EDM process resulted in a high surface hardness. However, the presence of titanium perovskite in the coating reduced gradually with an increased coating depth [153]. Preethkanwal et al. [154] explored the surface characteristics of the machined Ti alloy through the HAp added-EDM process. A decline in wear-rate was observed because of both oxide and carbide formation. The microhardness of a treated β-phase titanium alloy surface utilizing HAp in the EDM process was improved to 1275 HV [70]. A maximum microhardness value of 80.7 HRC was estimated due to the generation of ZrC, ZrO, CaTiO_3_, and TiC on the machined surface of Zr-based BMG when HAp was added to the working liquid of the EDM operation [71]. The wear-rate of a treated Mg alloy declined by 90.85% due to the formation of a HAp-based coating through the PM-EDM technique, which was 0.07 mm/year. The treated surface microhardness improved to 234 HV, i.e., 1.5 times greater than the parent material [155].

## 6. Influence of Corrosion Behavior on Biomaterial Performance

**Figure 6:**
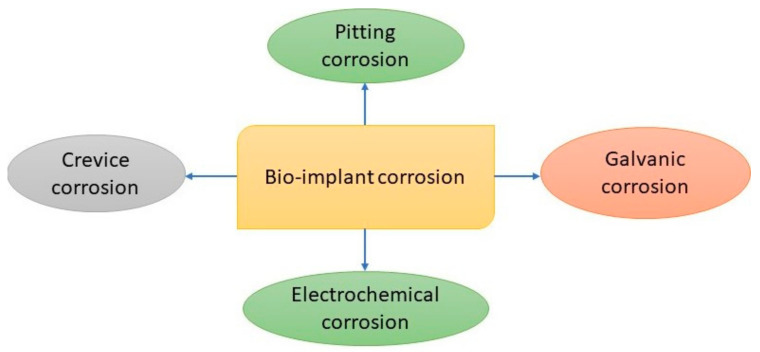
Basic types of corrosion behaviour induced in biomaterials.

Corrosion is basically an electrochemical process that is characterized as an irreversible material degeneration because of the chemical reactions occurring between the material and its environment. The corrosion behavior of metallic biomaterials is widely measured for quality assurance and failure analysis, because the functionality, sustainability, and biocompatibility of biomaterials rely on their corrosion behavior. It has been suggested that “the more corrosion resistance, the more biocompatible” [38,79,117,156]. Implanted biomaterials have been recently claimed to start physical decay within 12–15 years, caused by electrochemical reactions, although most metallic biomaterials have a high corrosion resistance [38,156,157]. Therefore, corrosion is regarded as an important factor for the designing and selection of biomaterials for biomedical applications. The inserted implants confront challenges regarding their corrosion behavior, owing to electrochemical reactions due to the presence of aqueous liquids in the body. Chloride, pH-levels, and dissolved-oxygen are the most significant factors of body fluids that affect the corrosion behavior of metallic implants. In body fluids, key cations contain ions such as potassium, sodium, hydrogen, magnesium, and calcium. On the other hand, important anions include the ions made of hydroxide, chloride, sulfate, bio-carbonate, and phosphate. The most influential factors that affect the corrosion behavior of all metallic implants are the dissolved salts. The temperature and pH level of the body fluids also affect the electrochemical process. An increment of the body fluid temperature stimulates electrochemical reactions, resulting in a high corrosion rate. A decline in the pH level of the surrounding body fluid may cause the localized corrosion of biomedical devices [38,115,158,159]. Figure 6 shows the basic types of corrosion behavior induced in biomaterials.

**Figure 7:**
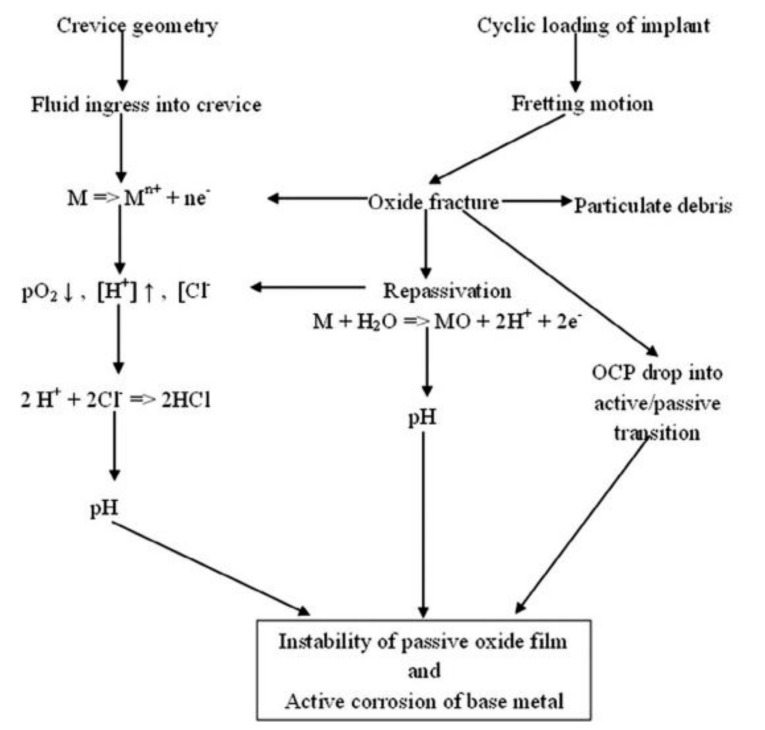
Mechanism of mechanically induced crevice corrosion in the implants (reproduced with permission from ref. [160].

The different types of corrosion mechanisms that are pertinent to recent metallic biomaterials include pitting, galvanic, uniform, stress cracking, crevice, fatigue, intergranular, and fretting corrosion. Figure 7 demonstrates the mechanism of mechanically induced crevice corrosion in implants.

**Figure 8:**
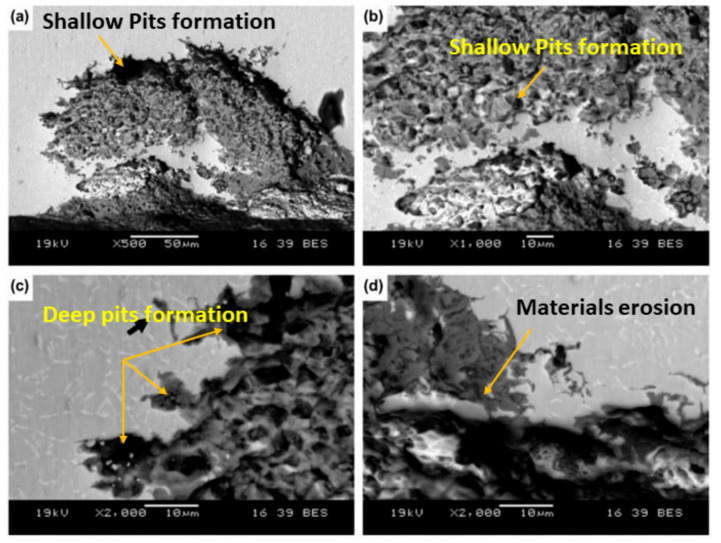
Effect of pitting corrosion on a neck stem device made from Ti-6Al-4V (**a**,**b**) showing pits formation, (**c**) displaying deep pits formation, and (**d**) material erosion due to corrosion (reproduced with permission from ref. [160]).

This Figure 7 illustrates the destruction of the passive oxide film and activation of corrosion when implanted bio-implants are experiencing cycling loads and crevice geometry. Due to oxide film fracture, the particulate debris are released into the body. Though the fractured film reforms the passive oxide film by reacting with body water, it is destroyed due to the pH conditions of the body fluids. Pitting corrosion is localized corrosion which occurs due to the presence of dissolved salts [115,120,157,160,161]. Figure 8 shows the impact of pitting corrosion on a neck stem device made from Ti-6Al-4V.

Figure 8a–d demonstrates the surface morphology when implanted biomaterials experience pitting corrosion. A destruction of the passive oxide film with shallow and deep pits is observed in Figure 8a–d. The quantity of the eroded elements caused by the corrosion can be calculated using Faraday’s Law [38]. Due to the occurrence of corrosion, debris such as Ni, Co, V, Al, Cr, and so on, eroded from the metallic implant’s surface, are released into the body causing alterations to cell performance, biological responses, a shortened lifecycle of the biomaterial, bone loss, implant loosening, toxicity, allergic effects, inflammation, and premature implant failure [15,64,159,162,163,164,165]. An excessive presence of Fe particles in the blood causes liver failure, long-term organ damage, and damage to lipids, DNA, and proteins [166,167]. High quantities of Cr and Co in the human body result in hemolysis and muscle fatigue [165]. In summary, the corrosion phenomena from metallic implants may have three effects on body tissues: (1) electrical currents can influence cell behavior, (2) altering the chemical environment, and (3) the metal ions can influence the metabolism of cells [38,168]. Table 4 summarizes the influence of released debris from biomaterial surfaces on biological responses after corrosion and wear occur inside a living body.

## 7. Importance of HAp-Based Coatings for the Corrosion Behavior of Biomaterials

To improve corrosion resistance, the introduction of surface coatings to biomaterials utilizing bio-ceramic nano-powders is considered an effective solution. The researchers who are working in this field have focused on developing several surface modification techniques to create superior bioactive surfaces and enhance the mechanical characteristics of biomaterials [157,163,169]. Sarbjit et al. [176] investigated the hardness and corrosion properties of treated 316L stainless-steel using both HAp and HAp/TiO_2_ with the high velocity flame spray technique. The results showed an increase in both the microhardness and corrosion resistance of the HAp-based coating, whereas the HAp/TiO_2_-based layer outperformed it in both areas. Dunne et al. [171] examined the impact of an HAp-based coating on the corrosion rate of an Mg alloy using the blast coating method. A low corrosion rate with crystalline phase was reported for the HAp coated surfaces, but not the uncoated surfaces. Hortensia et al. [172] conducted a comparative research work on the nature of the corrosion of HAp and TiO_2_-HAp-based coatings on a Ti alloy prepared through the high velocity oxygen fuel (HVOF) technique. The outcome exhibited damage to the HAp-based coating due to its dissolution when it was immersed in the simulated body fluid (SBF), but the addition of TiO_2_ particles to the HAp resulted in the creation of active protection against the corrosion. Gao et al. [173] explored the corrosion and bioactive properties of an Mg alloy which was modified through applying HAp in the plasma-spray technique. The modified surface provided a higher corrosion resistance and bioactivity compared to the uncoated Mg alloy. Durairaj et al. [174] synthesized a HAp-based layer on both a Ti alloy and an Mg alloy by the electrodeposition method. The degradation and corrosion rate were lower for both the treated alloys compared to the untreated specimens. The bio-growth of an Hap-coated Mg alloy was promising, but for an Hap-coated Ti alloy, the bio-growth was not significantly improved. However, the morphological tests after completing the corrosion experiments using the SBF verified the formation of multiple cavities and pores on the HAp-based coatings. The mechanism of pore formation during the potentiodynamic polarization technique with Hanks’ solution follows two processes: (i) the formation of H+ ions on the implant surface, and (ii) the acidification of the medium by producing H+ ions that dissolve the HAp and form larger pores [64]. Hence, to resolve these issues, researchers around the world have recently been attempting to amalgamate reinforcement agents such as CNT, Nb, Ag, Sr, Fe_3_O_4_, Si, Mg, and so on with HAp to prevent this manner of dissolution, which significantly improves the mechanical properties, including the microhardness, wear resistance, and corrosion resistance [39,174,175,177,178,179].

Chander et al. [70] explored the properties of treated Ti alloy surfaces prepared through the HAp added-EDM process. A formed biocompatible layer comprising oxides showed a higher corrosion resistance for the modified surfaces with excellent biocompatibility compared to the untreated specimens. In the research work by Preetkanwal et al. [154], a low corrosion value of 0.1146 mm/year was observed when machining a Ti alloy using a nano-HAp mixed dielectric in the EDM technique. Razak et al. [180] studied how to develop a formula for controlling the corrosion rate of an Mg alloy applying zinc powders in the EDM method. In this research work, corrosion values ranging from 0.000183 mm/year to 0.001528 mm/year were obtained when 2 g/L zinc was added to the working fluid of the system. Chander et al. [56] synthesized a nano-HAp-based coating on an Mg alloy through the PM-EDM method. In this research, the corrosion rate of the coated Mg alloy declined by 90.85%, ranging between 0.82 mm/year and 0.07 mm/year due to formation of a stable protective layer consisting of intermetallic compounds and oxides.

## 8. PM-EDM Process Variables Affecting the Treated Surface Properties

Due to the dynamic nature of the PM-EDM system, the interpretation of the interactions and effects of factors on the treated surface properties are difficult. The process factors are divided into the electrical and non-electrical factors on which the machined surface characteristics depend. Current, applied voltage, pulse-on duration, and pulse-off duration are the most influential electrical variables, while the non-electrical input variables include the physical properties of the tools, working liquid, particle size, and the amount of powder added [11,23,181,182,183,184,185,186,187,188]. Considering that a PM-EDM system is complex in nature, optimization of the process factors is required to obtain the optimum responses. A summary of the influence of process factors on the machined surface properties is given in Table 5.

### 8.1. Effect of Non-Electrical Parameters on the Modified Surface Characteristics

The amount of added powders and the size of particles are vital factors which influence the altered surface quality. A rise in layer thickness of the modified part is obtained at a high concentration of added powder, since the deposition rate rises during machining. However, a much thicker coating contributes to poor adhesion bonding, due to reducing the compressive stress in the coating, leading towards wear propagation. Moreover, both corrosion and microhardness were observed to be augmented with the subsequent mixing of more additive, due to the oxide and carbide formation during machining [9,11,74,191,192].

The dielectric fluid is considered another vital factor influencing the performance of the EDM process. Dielectric fluids, including EDM oil, mineral oil, kerosene oil, deionized water, and distilled water, are commonly employed in different EDM applications. Basically, micro-EDM and wire-cut EDM use deionized water and distilled water, whereas die-sink-EDM employs EDM oil, mineral oil, and kerosene. Deionized water and distilled water show some limitations when utilized for biomedical applications due to being prone to higher corrosion rates compared to mineral oil, EDM oil, and kerosene. Mineral oil, EDM oil, and kerosene provide a higher microhardness of the treated specimen than deionized water because of carbide formation. Furthermore, a thicker recast layer has been reported with application of oil-based dielectrics instead of water-based dielectrics, due to having a lower specific heat and higher electrical resistivity, decreasing the machining gap and poor flushing [57,191,193,194].

### 8.2. Effect of Electrical Factors on Modified Surface Properties

An increase in peak current provides a large amount of electrical energy in the system, which causes further fusing and vaporization. A high application of current induces further melting, migration, and deposition of the melted debris, resulting in the formation of a thick substrate during machining. Due to the surface alloying caused by the application of a high discharge current, a hard surface is observed. Moreover, the microhardness of the treated surface is elevated due to the accelerated heating and cooling processes at high current application, although surface alloys are not formed. A rise in the migration and deposition of the melted debris is displayed with high current application, confirming the carbide and oxide formation on the machined part, which contributes to enhancing the biocompatibility and corrosion and wear resistance; though micro-cracks, residual stresses, and a reduction of compressive stresses may be noticed [54,57,70,74,182,195]. Followed by the peak current, an increase of pulse-on duration supplies more electrical energy to the plasma channel due to applying sparks for a long period. Consequently, more materials from the employed tools, dielectric fluid, and added powders are eroded and deposited on the machined surface, causing hard and thick recast layers [53,182,196,197,198].

The applied voltage is from about 50 to 300 volts for the PM-EDM system because a high potential voltage is not appropriate for high-precision machining. The potential voltage affects the spark size and removal of the materials. An augmentation in coating thickness and deposition rate is found at high potential voltage applications, but these decline at too large a voltage application [57,182,199].

Pulse-off duration is another crucial electrical factor because its proper selection ensures stable machining. Although a long pulse-off duration is preferred due to allowing more flushing of the produced debris from the machining gap, it may cause overcooling of the eroded debris, resulting in a thick recast layer. On the other hand, the application of short pulse-off durations causes unstable machining, due to the occurrence of short circuits [11,182].

## 9. Current Surface Coating Techniques for the Biomedical Applications

HAp-based coating stability is a critical consideration for potential bio-implants, since phosphate–calcium is the most abundant substance in both bone and the teeth and produces bioactive responses. Various surface treatment methods that are currently used for coating purposes affect characteristics of the HAp-based layer including the mechanical strength, wear and corrosion resistance, and biological performance by processing the coating with a high sintering temperature [17,18]. A comparative study of HAp-based coating thickness, microhardness, and wear and corrosion resistance using various methods is presented in Table 6.

The methods of deposition are divided into two categories: chemical and physical depositions. The thermal spraying method, laser surface alloying, spray pyrolysis technique, sputtering process, pulse laser deposition, and laser melting deposition are the physical alteration techniques, while the chemical modification techniques include the following: sol-gel, hot pressing, dip coating, electrochemical method, electrostatic spray, and electrophoretic [17,18,19,48]. The plasma-spray coating method is widely used in the biomedical industry, synthesizing the Ca-P based coating on the biomaterials by spraying melted HAp and using an electric arc [15,200,210,211,212,213,214,215]. Gao et al. [173] explored the nature of corrosion and biological responses of an HAp-coated Mg alloy prepared through the plasma-sprayed technique. The HAp-based coating showed a low corrosion rate with excellent bioactivity due to the formation of β-Ca_3_(PO_4_)_2_, which is the converted form of the HAp. Moreover, the obtained coating was hydrophilic in nature. Although the coating deposition rate was comparatively higher in this process, a high deposition involves an intensive amount of heat application during the operation, resulting in alteration of the HAp alloying phases [211,212,216]. The sol-gel deposition process is another common technique for surface modification that follows a simple procedure and enables coating the complex shapes of metallic biomaterials [206,207,217,218,219]. Sarbjit et al. [205] synthesized a HAp-based layer on a 316L stainless steel through the sol-gel method. The obtained coating thickness of about 250 µm showed high corrosion resistance and excellent bioactivity. It has already been proven by previous studies that an improved hardness and corrosion resistance can be obtained by the sol-gel process, though the formation of a porous surface with this process can be very difficult to manage [220]. The biomimetic process can prepare a thick and porous coating with improved morphology compared to other techniques [221]. Shalinder et al. [170] made a comparison of HAp-based coatings formed through both the plasma spray and biomimetic methods. The plasma sprayed HAp method produced a thick coating and showed a higher microhardness and corrosion resistance compared to the biomimetic process. The dip coating technique follows steps such as dipping, withdrawal, and drying, and shows various advantages, such as low installation cost, ease of working, capability for complex coatings and shape, uniform layer, and low working temperature [208,222,223,224]. Faiz et al. [209] explored the characteristics of an HAp-based modified surface of a Ti alloy made using the dip-coating technique. The modified surface had a high corrosion resistance and biocompatibility. The electrochemical deposition (ECD) process operated at a moderate temperature is one of the coating techniques available in the biomedical industries that can form a homogeneous coating and perform rapid coating deposition [143,203,204,225,226,227]. Saadati et al. [202] prepared an HAp-based coating on Mg-4Zn-4Sn-0.6Ca-0.5Mn alloy using the electrophoretic coating technique. The obtained coating thickness of more than 100 µm showed an enhancement in corrosion resistance for the coated samples. Although both the physical and chemical coating methods have some advantages, they also have some limitations in the issues, such as coating process, coating strength, coating compounds, porous coating, wear and corrosion behavior, and cost of production [11,48]. A comparative study regarding the advantages and disadvantages of the existing coating methods with the PM-EDM technique is shown in Table 7.

## 10. Critical Analysis of the Effects of HAp on the Treated Surface Properties through PM-EDM

Regarding surface treatment of the widely employed biomaterials such as alloy steel, titanium alloy, cobalt alloy, and magnesium alloy using the PM-EDM processes, there are few literature studies available online as it is a newly developed trend in biomedical applications. In the research work by Gurpreet et al. [73], a high microhardness of 877.60 HV was calculated corresponding to a 28 A current, pulse-on period of 120 µs, and 15 g/L HAp concentration, which was improved by 160% compared to the untreated 316L steel. Thus, the HAp mixed dielectric medium showed a direct effect on the microhardness. Chander et al. [56] analyzed the surface properties of an Mg alloy, which was modified using the nano-HAp mixed-EDM technique. With the addition of HAp in the EDM process, the analysis demonstrated an improvement in corrosion resistance and microhardness of the treated surface by 90.85% and 1.5 times, respectively. Furthermore, due to the development of intermetallic oxides, an augmentation in the machined surface biocompatibility was noticed. In this study, a thick layer of 15–18 µm was formed at a HAp concentration of 20 g/L. However, the increase of HAp to 20 g/L may have resulted in the immediate settling of the HAp during machining [11], and a thick layer reduces the compressive stresses, resulting in inferior adhesion of the coating and wear resistance [48,74,87]. Chander et al. [53] analyzed the influence of the process factors on machined Mg alloy surface responses using the HAp added-EDM method. In this experiment, multi-objective particle swarm optimization (MO-PSO) was employed to optimize the related process variables, such as the HAp concentration, current, pulse-on duration, and pulse-off duration, thus, obtaining the optimum values for the machined surface properties, including the roughness, microhardness, and layer thickness. With an application of 5.28 g/L HAp, 3.48 A current, pulse-on duration of 40.33 µs, and pulse-off duration of 109.29 µs, the optimum performance with a 246 HV microhardness and 11.85 µm recast layer thickness was obtained. However, by employing a long pulse-off duration, a thick recast layer was created that may have led to the mechanical malfunction of the modified biomaterials [19,74,228]. With the addition of a small amount of HAp, the recast substrate thickness decreased, which then increased due to applying an HAp amount of more than 3–4 g/L [189]. Further increasing the HAp quantity to above 5 g/L confirmed the greater HAp presence in the machining void, followed by an improvement in deposition rate. The formation of carbides and oxides was observed with an increase in both the added-HAp and the current, resulting in an improved microhardness and corrosion behavior. Nonetheless, the presence of more hard carbides may be a source of wear propagation [87,229]. Preetkanwal et al. [154] explored the treated surface characteristics of a titanium alloy prepared utilizing the nano-HAp suspended-EDM technique. The surface modified using the HAp exhibited a decline in wear rate of 82% (68 µm) compared to the untreated sample and an increase in bioactivity due to the formation of TiO_2_, VSi_2_, (Ca_3_(PO_4_)_2_), TiC, and CaTiO_3_ with the nanopores. Furthermore, the HAp-based surface revealed a corrosion potential value of −0.0692 mV, resulting in a low corrosion value of 0.1146 mm/year, although the influences of the associated parameters on the wear and corrosion behavior were not illustrated. The obtained HAp-based coating thickness was not measured, which is considered an important factor for both the corrosion and wear behavior.

**Figure 9:**
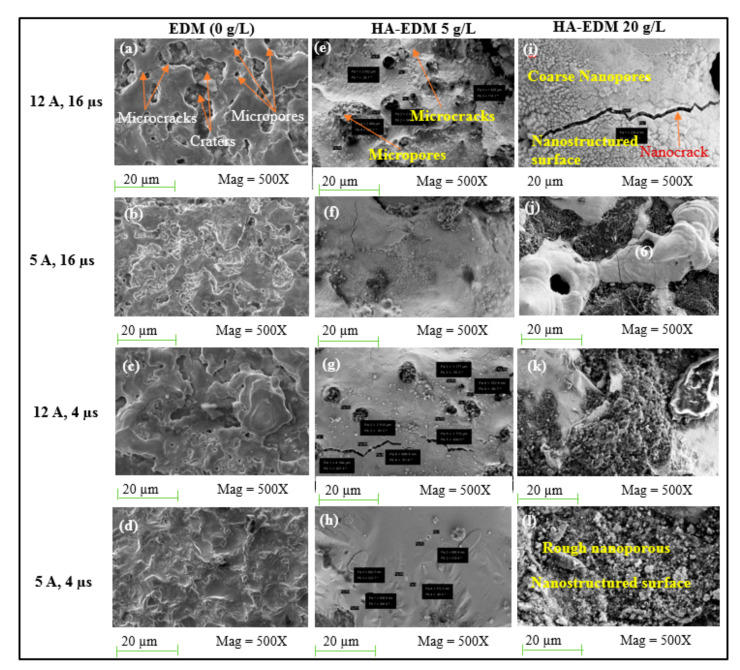
Influence of current and HA powder concentration on the modified surface morphology (**a**–**l**) showing microcracks, micropores, shallow craters, and nanostructure formation (reproduced with permission from ref. [54]).

Shih-Fu and Cong-Yu [189] the evaluated the impact of the HAp suspended-EDM process on the surface morphology of a treated titanium alloy. A thin layer of 9 µm was measured when mixing 5 g/L HAp in the dielectric liquid. A mathematical mode of the recast layer thickness was designed as a function of the HAp quantity, which was increased by adding a further 5 g/L of HAp. The treated surface microhardness was enhanced by three times compared to the untreated substrate, due to increasing the HAp concentration [189]. Chander and Uddin [70] analyzed the surface properties of a treated Ti alloy prepared using the HAp added-EDM technique. An increase of the HAp concentration improved the oxide and carbide deposition on the modified surface, resulting in a three-fold improved microhardness and high corrosion resistance and biocompatibility. The measured layer thickness of 7 µm at a HAp quantity of 5 g/L was smaller than the obtained coating thickness of 10 µm at a 0 g/L powder addition, although increasing the HAp concentration to 15 g/L augmented the thickness to 18–20 µm as greater trapping of the added powders resulted from shortening the gap distance. Aliyu et al. [54] synthesized a HAp-based coating on a zirconium-based bulk metallic glass (BMG) through the PM-EDM method. In this study, due to the formation of CaZrO_3_, ZrO_2_, Ca_5_(PO_4_)_3_OH, TiC, and ZrC, the modified surface exhibited an increase in both hardness and biocompatibility. The coated surface revealed a coating thickness of approximate 27.2 µm with an increase in the added powder concentration and a decrease in the discharge energy. The influence of the current, pulse-on duration, and HAp concentration on the surface morphology of the machined Zr-based BMG is shown in Figure 9.

Figure 9 shows the formation of nanopores, shallow craters, and microcracks on the coated BMG surface through the HAp mixed-EDM process at different machining conditions. A nanoporous and lakargiite (CaZrO_3_)-based coating on BMG was synthesized by Aliyu et al. [9], while mixing HAp in the dielectric. Owing to the formation of ZrO, ZrC, TiC, and CaTiO_3_, the microhardness of the treated surface was improved by around 42%. An increase in microhardness of the modified surface of the bulk metallic glass was found in this research when applying a low current and both a high pulse-on time and powder concentration in the process. Alamin et al. [74] synthesized a uniform thin recast layer on a modified 316L steel surface using HAp mixed-EDM process, which is crucial for enhanced corrosion resistance and biocompatibility. In this study, uniform coatings were obtained corresponding to all the parameters settings, which were achieved for the first-time using the HAp added-EDM process. A largest coating thickness of 15.294 µm was obtained, corresponding to a peak current of 10 A, pulse-on duration of 16 µs, and HAp concentration of 15 g/L, whereas the lowest coating thickness of 6.22 µm was recorded at a 5 A peak current, 8 µs pulse-on duration, and 10 g/L HAp amount. Table 8 reports a summary of the current developments of obtained layer thickness, microhardness, wear and corrosion behaviours using the HAp mixed-EDM process.

## 11. Challenges, Followed by Areas for Future Research

Nowadays, there are few published works available which provide a clear conceptualization regarding the mechanism of recast layer formation using the PM-EDM process. As a matter of fact, the modified surface properties, such as a uniform thickness of coating, phases of coating, microhardness, and wear and corrosion behavior using the HAp mixed-EDM technique have not been elaborated in detail because few studies have been produced over the last few decades. Furthermore, few research has showed the correlation between the associated factors of the PM-EDM process and the machined surface properties, resulting in a lack of knowledge regarding these aspects. Hence, these aspects should be investigated in detail.

There are so many research areas remaining regarding the HAp suspended-EDM process. Researchers are focusing on the HAp-based coating thickness, Ca-P ratio, and phase transformation during the machining. The challenge is to achieve a uniform thin recast layer with homogeneous alloying, which leads to a rise in the compressive stresses and a decline in the residual stresses. It is also very challenging to ensure a proper distribution of the produced oxides and carbides on the machined part. Moreover, a large amount of heat is generated during the PM-EDM operation, resulting in phase transformation of the HAp and the HAp-based alloys, which leads to a poorly modified surface quality regarding the mechanical characteristics, biological response, and tribo-corrosion behavior. To ensure a proper distribution of the added substances in the coating and enhance the mechanical properties of the HAp-based layer, the HAp should be doped with other additives; leading to another challenge during the machining, as the doped HAp contributes a high molecular weight, resulting in a difficult to prevent quick-settling tendency. Furthermore, a proper combination of crystallinity and amorphous phases in the coating should be ensured.

The corrosion behavior of biomaterial is another important concern on which the stability and biological responses rely. It has been confirmed from the literature that the HAp-based coating may dissolve in an SBF environment, which leads to the addition of the reinforcement additives to the HAp to improve the corrosion resistance. Therefore, proper selection of additives is another challenge for future researchers. Furthermore, due to applying a high discharge energy during machining and having a low heat conductivity of the HAp, micro cracks develop in the HAp-based coating after solidification and the crystallinity of the added-HAp may be changed, resulting a high corrosion rate.

**Figure 10:**
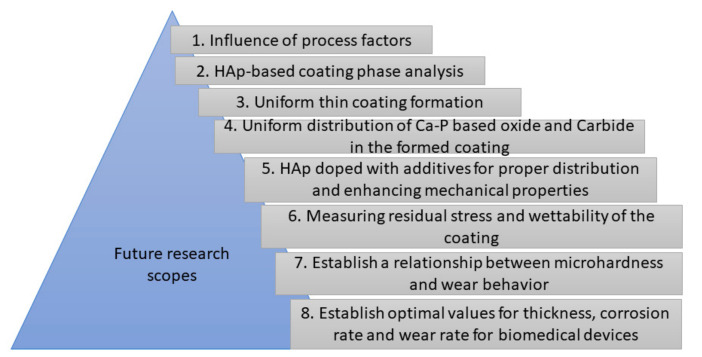
Summary of future research areas.

The wear behavior of biomaterials is regarded as an important mechanical property, which may cause mechanical failure of inserted implants. This property may depend on the design, manufacturing process, coating surface quality, coating alloying, and so on. During the PM-EDM process, a very high temperature, ranging from 8000 °C to 12,000 °C, is created, which may cause the creation of residual stresses in the coating. These stresses may make the implant surface prone to wear. Estimating the residual stresses from the developed layer is another challenge. The surface roughness and the wettability of modified layer are important factors for the introduction of wear. The challenge lies in obtaining the proper roughness and wettability of the modified surface to improve the wear resistance. Presently, there is no established mathematical relation between the microhardness and the wear behavior. Therefore, a clear relationship between them should be established to resolve this issue. Figure 10 summarizes future research areas regarding HAp-based coating using the PM-EDM process.

To resolve the aforesaid limitations of HAp-based coating, a suitable additive, having a high electrical and heat conductivity, should be added to the HAp mixed dielectric fluid during the EDM process to act as a reinforcement agent. Carbon nanotubes (CNT) yielding graphene (C) are recommended as a suitable additive and are considered the strongest materials in the world nowadays. The CNT offers outstanding mechanical, thermal, and electrical properties. For instance, it is 100 times stronger compared to steel alloys. Moreover, it has a high Young’s modulus of 1 TPa, a strength to weight ratio 500 times greater than aluminum, and a strain 10% higher than any other material. It offers a higher electrical conductivity, at 109 A/cm^2^, than that of copper, at 106 A/cm^2^. It even possess a high thermal conductivity of 3320 W/mK [230,231]. From a biocompatibility point of view regarding CNT, most studies delivered positive feedback, although a few investigations suggested checking its cytotoxicity before using it in the biomedical applications, because of the presence of Ni, Fe, and Al in CNT [232,233]. As the CNT has a small specific gravity and larger dynamic shape factor than a spherical shape [59], it can uniformly mix with EDM oil and disperse through the machining gap, resulting the removal of microcrack formation from the HAp-based coatings caused by uniform heat transfer during the cooling process. Moreover, a thin, uniform coating may be formed using CNT due to improving flushing of the produced debris and their scouring effects.

## 12. Summary

The biological functions and the characteristics of modified surface are proposed as the key factors for long-term implementation of bio-implants in the human body. HAp-based coatings have been required to ensure the biocompatibility and the biofunctions of biomaterials, which results excellent osseointegration and faster growth of the hard tissues. Furthermore, in previous literature it has been reported that the treated surface characteristics, such as the coating thickness, microhardness, and wear and corrosion resistance were improved using the HAp added-EDM process. However, the literature reported the dissolution of HAp-based coatings in the SBF solution, leading to the incorporation of other additives with HAp. Though the existing coating techniques have some advantages, the PM-EDM technique shows some advantages over the conventional methods, as it is (a) able to form and coat concurrently; (b) does not require preparing the surface before machining; (c) capable of forming carbides and oxides on the machined part; (d) capable of forming a nano-porous layer; and (e) able to improve the surface microhardness and wear and corrosion resistance; and thus may be a potential candidate in the field of processing biomedical devices.

Based on the recently published research works, this article provides a critical analysis of the effects of HAp addition to the EDM process and the associated process factors on the surface characteristics of biomaterials. This study thoroughly highlights the PM-EDM process mechanism and the importance of HAp for enhancing the biological responses and the surface properties, such as the coating thickness, microhardness, and wear and corrosion behavior. Furthermore, a comparative study of the modified surface characteristics using both the HAp added-EDM method and other existing methods was critically reported. In this review, the feasibility of the HAp mixed-EDM process for biomedical applications was compared with the other conventional coating techniques. A machined surface morphology and topography were depicted to validate the analysis. A few literature studies regarding HAp-based coating phase transformation and the wear and corrosion behavior using the PM-EDM method have recently been produced, which contribute to the research areas for future researchers.

## Figures and Tables

**Table 1:** Mechanical characteristics, biocompatibility, and corrosion resistance of commonly used biomaterials [11].

Biomaterials	Density (gm/cm^3^)	Yield Strength (Mpa)	Tensile Strength (Mpa)	Elongation to Break (%)	Elastic Modulus (Gpa)	Biocompatibility	Corrosion Resistance
Biodegradable materials:							
Pure Mg	1.74–2	65–100	90–190	2–10	41–45	Excellent	Poor
AZ31 (Mg-alloy)	1.78	185	263	15–23	45		
AZ-91 (Mg-alloy)	1.81	160	150	2.5–11	45		
WE43 (Mg alloy	1.84	170	220	2–17	44.2		
Fe20Mn alloy	7.73	420	700	8	207		
Fe35Mn alloy	6.36	230	430	32			
Zn-Al-Cu (Zn based alloy)	5.79	171	210	1	90		
Non-biodegradable materials:							
316L steel	7.9	190	490	40	200	Poor	Moderate
Ti-6Al-4V	4.43	880	950	14	113.8	Fair	Excellent
Ti-6Al-7Nb	4.52	800	900	10	105	Fair	Excellent
CoCr20Ni15Mo7	7.8	240–450	450–960	50	195–230	Poor	Excellent
Other natural and synthetic materials:							
Synthetic HA	3.15		40–200		70–120	Excellent	Poor
Alumina ceramic	4		400–580	0.12	260–410	Excellent	Fair
Collagen			2.6–600	7.4–26.47	5–11.5	Excellent	Poor
PLGA	1.30–1.34	3.8–26.6	13.9–16.7	5.7		Excellent	Poor
PLC	1.145	8.37–14.6	68.45–102.7	22.8–28.3	281–686	Excellent	Poor

**Table 2:** Hydroxyapatite powder (HAp) phase transformation with increasing temperature during electrical discharge machining (EDM).

Temperature Range	Phase Transformation	Author
25–600 °C	Vaporization of liquid absorbed (oil or deionized water) during the process	[11,58]
600–800 °C	Decreasing carbon presence on the powder surface	
800–900 °C	Hydroxy-depleted layer covers the HAP which forms oxyapatite (OA)	
950–1400 °C	Hydroxyapatite is decomposed and converted into β-TCP (tricalcium phosphate) and TTCP (tetra calcium phosphate)	
1120–1470 °C	Tricalcium phosphate is changed to α-TCP which is stable at high temperature.	
1550–1630 °C	This is the melting point of hydroxyapatite and tetra calcium phosphate is still stable	
1650 °C	TTCP is melted and transferred into Cao compound. TCP is still stable	
1730 °C	TCP is melted	
>1750 °C	Amorphous calcium phosphate (ACP) starts to form	

**Table 3:** HAp coating requirements approved by the food and drug administration (FDA) [11].

Factors	Requirement
Density	2.98 g/cm^3^
Heavy metals	<50 ppm
Ca-P ratio	>1.67
Phase purity	>95%
Crystallinity	>62%
Coating thickness	not specified yet
Shear strength	>22 MPa
Tensile strength	>50.8 MPa

**Table 4:** Influence of released debris from biomaterials on biological responses during corrosion and wear.

Biomaterials	Application	Released Debris	Effects	Ref.
316L SS	1. Entire hip replacement	Cr, Ni, Co, Fe, and Mo	1. Altering cell performance	[167,168,169,170,171,172,173,174,175]
	2. Supporting devices (plates and screw)		2. Allergic effects	
Pure Mg	1. Biodegradable implants for orthopedic	Mg (no toxic effect)	3. Inflammation due to toxicity	
Mg alloys		Zn, Mn, and Ca (no toxic effects)	4. Immature implant failure	
Cobalt alloys	1. Full joints replacement	Ni, Co, and Cr	5. Bone loss	
	2. Dental implants		6. Implants loosening	
Ti alloys	1. Cup and stem of total hip replacement	Al, Ti, V, Mo, and Fe	7. Liver failure and organ damaged	
	2. Different fixed devices		8. Hemolysis and muscle fatigue	
Ni-Ti	1. Surgical instruments	Ni and Ti	9. Revision of surgery	
	2. Bone plates and Stents		10. Damaged of DNA and proteins	

**Table 5:** Summary of the effects of the process factors on the treated surface properties.

Biomaterials	Electrode	Added Powder	Effects on Modified Surface Properties	Ref.
Ti alloys	Copper	HAp	Wear resistant increased by 82% with increasing HA powder concentration.	[154]
	Graphite	HAp/CNT	Surface integrity improved while applying a higher current	[72]
	Pure Ti	HAp	A coating thickness of 9 µm with microcracks, three times higher microhardness, and Ca:P of 13 were obtained with a lower addition of HA powder.	[189]
	Pure Ti	HAp	A three times higher microhardness, higher corrosion resistance and biocompatibility, and a coating of 7 µm with crack free surface were observed with greater powder addition and current application	[70]
Zr-based alloy	Pure Ti	HAp	A thickness of approximately 27.2 µm, a higher deposition rate, and higher microhardness and biocompatibility were measured with increasing HAp quantity and decreasing discharge energy	[54]
	Pure Ti	HAp	Lakargiite at 50% and a rise of microhardness to around 42% were observed with a lower current and higher pulse on time and powder concentration.	[9]
316L SS	Copper	HAp	Biocompatibility was enhanced due to oxide and intermetallic alloy formation with higher discharge current	[190]
	Copper	HAp	Microhardness increased by 160%, bioactive alloys were found.	[73]
Mg alloys	Mg-Ca	HAp	Microhardness and wear resistant were enhanced by 1.5 times and 90.85%, respectively, biocompatibility increased significantly due to oxide formation, and coating thickness improved with increased powder addition	[56]
	Mg-Ca	HAp	Deposition rate increased with more powder addition, initially RLT decreased, while it increased with greater addition of HAp.	[53]

**Table 6:** Comparative study on HAp-based coating thickness, microhardness, wear and corrosion resistance through different surface modification methods.

Method	Materials	Layer Thickness	Microhardness	Corrosion Resistance	Wear Resistance	Ref
PM-EDM	Ti alloys	18–20 µm	Increased by 3 times	Increased	NM	[70]
	Ti alloys	NM	NM	Increased	Reduced by 82%	[154]
	Mg alloy	15–18 µm	Increased by 150%	Increased by 90.85%	NM	[56]
Plasma spray	Mg alloy	9 µm	NM	Increased	NM	[171]
	Ti alloys	NM	277 HV	Increased	NM	[200]
	Ti alloys	NM	339 HV	Increased	NM	[170]
	Ti alloys	185–200 µm	137 HV	Increased	NM	[201]
Electrophoretic	Mg alloy	70 µm	NM	Increased	NM	[202]
	Mg alloy	25–40 µm	NM	Increased by 30.84%	NM	[203]
	NiTi	7 µm	NM	Increased by 50 times	NM	[204]
Sol-gel	316L SS	250 µm	NM	Increased	NM	[205]
	Mg alloy	10.2 µm	NM	Increased by 40 times	NM	[206]
	316L SS	1.6 µm	459 HV	Less increased	NM	[207]
Dip coat	Ti	30–40 µm	232 HV	NM	NM	[208]
	Ti alloys	17.52 µm	NM	Increased	NM	[209]
Biomimetic	Ti alloys	Thin	327 HV	Less increased	NM	[170]

Note: NM-Not mentioned.

**Table 7:** Comparative study between commonly used coating techniques and the PM-EDM method [11,19,48].

Method	Layer Thickness	Advantages	Disadvantages
Dip coating	2–0.5 µm	Lower processing cost; possible complex shape coating with porous facility; quick deposition	Crack formation due to employing elevated sintering temperature; amorphous CaP coating formation due to mismatch between heating and cooling
Sol-gel	0.1–0.8 µm	Lower processing cost; possible complex shape coating with higher purity; lower operating temperature; higher cell adhesion	Expensive raw materials; low wear resistance due to higher porosity formation; higher permeability
Plasma spraying	30–200 µm	Less cost; higher coating rate; reduced risk of coating degradation; easy to operate	Non uniformity in coating density; Phase’s transformation due to high temperature; relatively lower adhesion strength; rapid cooling causes cracks; producing amorphous composites; synchronous establishment of biological agents is impossible
PM-EDM	3–65 µm	Can shape and coat simultaneously; can create porous and biocompatible coatings; higher adhesion strength; excellent corrosion and wear resistance; crack free surface possible; can obtain higher precision machining; inexpensive; excellent biological responses; research on-going process.	Can only cut conductive materials; cracks and craters are observed due to the high intensity of energy and poor flushing; lower machining efficiency; HA phases may be transformed due to elevated temperature; difficult to understand the deposition process; challenging to obtain uniform coating thickness; research on-going process.
Micro-arc oxidation	3–30 µm	Easy to control; inexpensive; environmentally friendly process; can coat complex geometries	Proper electrolytes required; considered a pre-deposition technique
High-velocity-oxy fuel spraying	30–200 µm	High coating rates; enhanced wear and corrosion resistance and biocompatibility	High temperatures cause non-stoichiometric and amorphous compounds; simultaneous incorporation of biological agents is impossible; crack propagation; line of sight technique
Electrochemical deposition	0.05–0.5 mm	Complex coating shapes possible; lower cost to operate; significantly uniform layer thickness possible	Poor coating adhesion strength; developing stress in coatings; difficult to control parameters
Pulse laser deposition	0.05–5 µm	Layers with amorphous crystalline phases; can produce an extensive range of multi-stages substrates by different materials; thick and porous substrates with excellent adhesive strength	Line of sight process; expensive; elevated temperature prevents simultaneous incorporation of biological factors; lack of uniformity

**Table 8:** Current status of layer thickness, microhardness, and wear and corrosion resistance using the HAp-mixed PM-EDM process.

Biomaterial	Layer Thickness	Microhardness	Corrosion Resistance	Biological Responses	Wear Resistance	Ref
Zr-based alloy	27.2 µm	Increased	NM	Increased	NM	[54]
	NM	Increased by 42%	NM	Increased	NM	[9]
Ti alloys	18–20 µm	3-fold increased	Increased	Increased	NM	[70]
	9 µm	3-fold increased	NM	Increased	NM	[189]
	NM	NM	Increased	Increased	Decreased by 82%	[154]
	20–50 µm	NM	NM	NM	NM	[55]
Mg alloys	11.85 µm	246 HV	Increased	Increased	NM	[53]
	15–18 µm	Increased by 1.5 times	Increased by 90.85%	Increased	NM	[56]
316L SS	NM	Increased by 160%	NM	Increased	NM	[73]

## Data Availability

Not applicable.
